# Colourfulness as a possible measure of object proximity in the larval zebrafish brain

**DOI:** 10.1016/j.cub.2021.01.030

**Published:** 2021-03-08

**Authors:** Philipp Bartel, Filip K. Janiak, Daniel Osorio, Tom Baden

**Affiliations:** 1 School of Life Sciences, Sussex Neuroscience, University of Sussex, Falmer, Brighton, UK; 2 Institute of Ophthalmic Research, University of Tübingen, Tübingen, Germany

## Abstract

The encoding of light increments and decrements by separate On- and Off- systems is a fundamental ingredient of vision, which supports edge detection and makes efficient use of the limited dynamic range of visual neurons^1^. Theory predicts that the neural representation of On- and Off-signals should be balanced, including across an animal’s visible spectrum. Here we find that larval zebrafish violate this textbook expectation: in the zebrafish brain, UV-stimulation near exclusively gives On-responses, blue/green stimulation mostly Off-responses, and red-light alone elicits approximately balanced On- and Off-responses (see also references^2^, ^3^, ^4^). We link these findings to zebrafish visual ecology, and suggest that the observed spectral tuning boosts the encoding of object ‘colourfulness’, which correlates with object proximity in their underwater world^5^.

## Teaser

Bartel *et al.* probe the bulk spectral tuning of the larval zebrafish visual brain using mesoscale two-photon imaging. They find a strong wavelength dependence of response polarity: UV-responses are nearly exclusively On, green/blue responses are mostly Off, and only red responses show an approximate balance of On and Off.

## Main text

**Figure 1:**
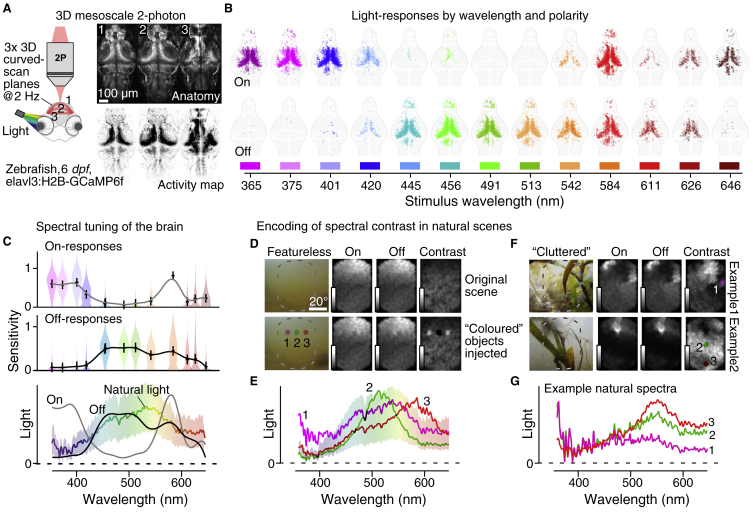
Spectral tuning of the larval zebrafish brain in the context of natural scenes. (A) Left, larval zebrafish expressing GCaMP6f in neuronal somata were imaged on a custom volumetric mesoscale two-photon system with three-dimensional multi-plane-bending to follow the brain’s natural curvature (described in reference^6^). Visual stimulation was by three second flashes of widefield light in 13 spectral bands (described in reference^10^). An example brain-wide quasi-simultaneously acquired tri-plane scan average (right, top) is shown alongside a projection of pixel-wise activity-correlation (right, bottom; dark indicates higher correlation). See also Figure S1. (B) x–y superposition of all On- and Off-responsive ROIs (top and bottom, respectively) across n = 90 planes from n = 13 fish to flashes of light at the indicated wavelengths. (C) Mean On- and Off-tuning functions based on (B), with crosses showing the median, and violin plots summarising the spread in the data at each wavelength (top, middle), and both tuning functions superimposed on the mean±SD availability of light in the zebrafish natural habitat (data from reference^8^). (D–G) Selected natural visual scenes from reference^8^, in each case showing an indicative photograph of the scene, followed by the full hyperspectral image as seen through the On-, Off- and On-Off-contrast filters (D,F) and associated full spectra (E,G), as indicated. The bottom panels of D are identical to the top with the addition of artificially ‘injected’ local spectral distortions as indicated in E to mimic, from left to right, a ‘UV-’, ‘green-’, and ‘red-object’. Grey scalebars are 0–0.6 (black to white) for On- and Off-reconstructions, and 0–0.02 for contrast-reconstructions.

To begin, we measured high-acuity spectral sensitivities of larval zebrafish brain neurons by two-photon imaging, capturing n = 11,967 regions of interest (ROIs) across the brains of n = 13 six to seven day post-fertilization zebrafish (elavl3:H2B-GCaMP6f; Figure 1A and Figure S1A–C). To record the entire brain along its natural three-dimensional curvature we used a non-telecentric mesoscale approach coupled with ‘intelligent plane bending’ enabled by rapid remote focusing^6^ (Video S1 and Figure S1A). A custom hyperspectral stimulator consisting of 13 spectrally distinct LEDs opposing a diffraction grating and collimator for collection^7^ allowed wide-field stimulation, which was approximately aligned with one eye’s retinal acute zone. Regions of interest corresponding to individual and/or small groups of similarly responding neuronal somata were extracted from each recording, then quality filtered, denoised and decomposed into On- and Off- responses (Figure S1A–G and Supplemental Experimental Procedures).

Recordings revealed that, despite some expected variation^2^, ^3^, ^4^ (for example, Figure S1B), neural responses in all major visual centres of the brain had a common, overarching spectral sensitivity profile: UV-On, Blue/Green Off, Red On-Off (Figure 1B). This organisation into three spectral processing zones (UV, Blue/Green, Red) can be linked to visual ecology. First, the UV On- responses likely serve prey-capture of aquatic microorganisms such as paramecia, which appear as UV-bright objects when illuminated by the sun^7^. Second, the approximate balance of red On- and Off- responses may allow zebrafish to use the abundance of long-wavelength illumination in shallow water^8^ to drive ‘general-purpose’ achromatic vision, including motion circuits^9^. Third, the dominance of Off responses to blue and green wavelengths may serve as a subtraction signal to spectrally delineate the red- and UV-systems^2^, and to provide a spectral opponent signal for colour vision against UV- and red-On circuits^10^.

Video S1. Two-photon curved triplane mesoscale imaging of the larval zebrafish brain during hyperspectral full-field stimulation | related to Figure 1A and Figure S1A-C Combined responses of one zebrafish’s brain to flashes of different wavelengths of light presented in sequence (cf. Figure 1B, Figure S1D) based on three consecutive scans with three planes each (cf. Figure S1A). Data is averaged over 4 stimulus loops and sped up to 5x real time. In the second video segment, the central panel from the first segment is isolated and montaged to display responses to all 13 tested wavelengths in synchrony, as indicated.

A further non-mutually exclusive interpretation is that spectral organization in the zebrafish brain accentuates ‘colourfulness’, which could act as a cue to object proximity. This is because unlike air, turbidity in aquatic environments rapidly attenuates both achromatic and chromatic contrasts with distance^5^, so that any high-contrast and/or colourful underwater object must be nearby.

To explore this idea, we computed the mean zebrafish brain On- and Off-spectral sensitivities and compared them to the average availability of light in the zebrafish natural habitat^8^ (Figure 1C). This revealed a good match between natural spectra and the brain’s Off-filter, whereas the On-filter sensitivity peaked beyond the range of highest light availability. Nevertheless, the generally positive rectification of brain responses (Figure S1D,E,G) meant that both the Off- and the On-filter signals strongly correlated with brightness (Figure S1J,K). Accordingly, either filter in isolation encoded achromatic information, which dominates natural scenes. This correlation, however, also meant that, when computing On-Off contrast (On–Off)/(On+Off) as a function of wavelength, brightness information was essentially cancelled to instead highlight spectra that differed from the mean — chromatic information (Figure S1L).

To illustrate how such an On-Off contrast filter would serve to highlight ‘colourfulness’ in nature, we reconstructed individual natural scenes from hyperspectral images. In each case we computed three reconstructions: On-filter alone, Off-filter alone, and On-Off contrast (Figure 1D–G). In a featureless scene along the open water horizon, both the On- and Off-reconstructions were dominated by the vertical brightness gradient, while the On-Off reconstruction showed approximately homogeneous activation (Figure 1D, top). We then artificially skewed the underlying spectra of three neighbouring regions in the same image to mimic small UV-, green- and red-biased objects, respectively, and again computed the On-, Off- and On-Off representations (Figure 1D, bottom, cf. Figure 1E). This manipulation had only minor effects on the On- or Off-reconstructions, but the contrast reconstruction readily reported the presence of all three objects. Similarly, On-Off contrast reconstructions lent themselves to reporting foliage in the foreground in non-manipulated, cluttered natural visual environments (Figure 1F,G).

Together, our data suggest that the zebrafish brain’s overall spectral On-Off tuning is suited to represent the presence of spectral information that differs from the mean, and thus to provide a cue to object ‘colourfulness’, which in turn correlates with object proximity^5^. Beyond this overarching spectral response profile, substantial additional spectral diversity exists at the cellular and neurite levels, presumably to support the zebrafish’s various visual requirements^2^, ^3^, ^4^.

## Supplemental information

Document S1. Experimental Procedures and One Figure
