# Supplementary material for: Colourfulness as a possible measure of object proximity in the larval zebrafish brain
Source: Curr Biol. 2021 Mar 8;31(5):R235–6. doi: 10.1016/j.cub.2021.01.030 (PMC7955152; doi:10.1016/j.cub.2021.01.030)
Supplement: Supplementary file 2 — Document S1. Experimental Procedures and One Figure [file mmc1.pdf]

Supplemental Information

Colourfulness as a possible measure of object proximity in the larval zebrafish brain

Philipp Bartel, Filip K Janiak, Daniel Osorio and Tom Baden

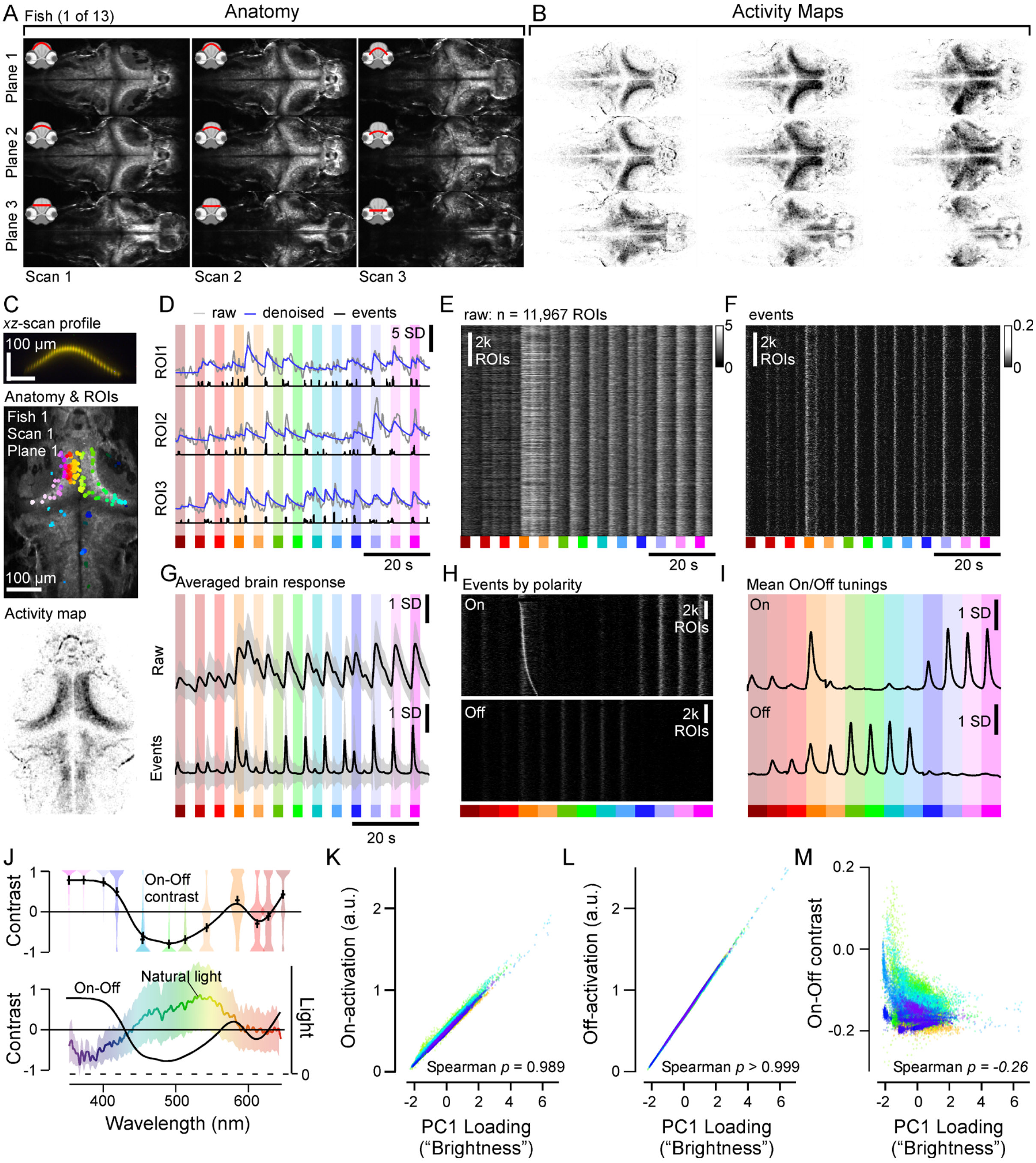

Figure S1. Measuring the bulk spectral tuning of the larval zebrafish brain. A, Example recordings from one larval zebrafish, comprising three consecutive scans of three planes each for a total of nine planes. For each triplane scan, starting from a common z-position,

the first two planes were bent upwards by ~100 µm and ~50 µm at the apex, respectively. The lowermost plane was kept flat. Between scans, the entire triplane was moved down by ~50 µm. In total, we recorded from n = 13 fish in such a configuration. **B**, Pixel-wise activity-correlation over time with the four neighbours computed as in Ref[S1] as an indication of locally correlated activity in the scan (darker shade indicate higher correlation). **C**, Example ROI extraction shown for plane/scan/fish 1 (cf. A,B), with xz-scan-profile visualised as in Ref[S2] (top), a crop of the anatomical projection with ROIs (middle) and corresponding activity map (bottom). **D**, Example ROIs from (C) in response to light-flashes of different wavelength as indicated, shown as z-normalised fluorescence (grey), denoised (blue) and detected events (black). **E,F**, All n = 11,967 ROIs from 13 fish (30 scans) shown as raw fluorescence (E) and as events (F). Note polarity switches between light-flashes of different wavelengths. **G**, Mean±1SD z-normalised fluorescence (top) and events (bottom) of all ROIs. **H,I**, Separate On- (top) and Off-event phases (bottom) extracted from (F,G) as heatmap (H) and mean tuning (I). All ROIs are sorted by the timing of the On-event in response to 584 nm ("peak" orange/red) light stimulation. In all heatmaps showing ROIs, lighter colours indicate a higher signal. **J**, as Fig. 1C, but showing On-Off contrast (i.e. the "brain filter"). **K-M**, Activation of the On- (J), Off- (K) and On-Off Contrast-filters (L) for each of 30,000 individual natural spectra (from n = 30 scenes[S3]) plotted against their "brightness", here computed as their loading against the first principal component (PC) that emerges from PCA across the entire dataset (see also Ref[S3]). Data from individual scenes is indicated by their different coloration. Spearman correlation coefficients ρ as indicated.

SUPPLEMENTAL EXPERIMENTAL PROCEDURES

RESOURCE AVAILABILITY

Lead Contact. Further information and requests for resources and reagents should be directed to and will be fulfilled by the Lead Contact, Tom Baden (t.baden@sussex.ac.uk).

Data and Code Availability. Pre-processed functional 2-photon imaging data and associated summary statistics are freely available at https://datadryad.org/stash/dataset/doi:10.5061/dryad.4xgxd2584 and via the relevant links on http://www.badenlab.org/resources and http://www.retinal-functomics.net. The natural imaging dataset was published previously as part of Ref[3].

EXPERIMENTAL MODEL AND SUBJECT DETAILS.

Animals. All procedures were performed in accordance with the UK Animals (Scientific Procedures) act 1986 and approved by the animal welfare committee of the University of Sussex. For all experiments, we used 6-7 *days post fertilization* (*dpf*) zebrafish (Danio rerio) larvae. The following previously published transgenic line was used: Tg(elavl3:H2B-GCaMP6f); ZFIN ZDB-ALT-150916-4[S4]. Animals were housed under a standard 14:10 day/night rhythm and fed three times a day. For 2-photon *in-vivo* imaging, zebrafish larvae were immobilised in 2% low melting point agarose (Fisher Scientific, BP1360-100), placed on a glass coverslip and submerged in fish water.

Light Stimulation. With fish mounted upright, light stimulation was delivered as wide-field flashes from a spectrally broad liquid waveguide with a low NA (0.59, 77555 Newport), positioned next to the objective at ~45˚. The other end of the waveguide collected light from 13 “spectrally narrowed” LEDs, as described in detail elsewhere[S5]. All stimuli were series of single LED flashes of light lasting 3 s, separated by gaps of 3 s (1 stimulus loop: 13 LEDs * (3+3) s = 78 s. 3-4 loops were presented and averaged for each recording.

2-photon calcium imaging. All 2-photon imaging was performed on a MOM-type 2-photon microscope (designed by W. Denk, MPI, Martinsried; purchased through Sutter

Instruments/Science Products) equipped with a mode-locked Ti:Sapphire laser (Chameleon Vision-S, Coherent) tuned to 960 nm for SyGCaMP imaging. We used one fluorescence detection channel (F48x573, AHF/Chroma), and a water immersion objective (W Plan-Apochromat 20x/1,0 DIC M27, Zeiss). For image acquisition, we used custom-written software (ScanM, by M. Mueller, MPI, Martinsried and T. Euler, CIN, Tuebingen) running under IGOR pro 6.3 for Windows (Wavemetrics).

To expand the field of view to ~1.2 mm diameter, which allowed capturing the entire brain's length in a single scan, we used a non-telecentric optical approach as described in detail elsewhere[S2]. The excitation spot (point spread function) in this configuration was ~0.7 µm (xy) and ~11 µm (z) at full width half maximum. This optical configuration can in principle capture the signals from individual larval zebrafish somata[S2]. However, in this work it was our intention to capture the bulk spectral responses across large fractions of the brain. Accordingly, we balanced recording area and spatial sampling such that individual somata effectively corresponded to single, or at most groups of 2-4 pixels (3 planes covering ~450x1,000 µm with a 160x350 px scan each to yield ~2.9 µm voxel xy-spacing, compared to average zebrafish neuronal soma diameter of ~7 µm; 1 ms per line, 2.08 Hz volume rate).

To follow the brain's natural 3D curvature, we also systematically 3D-bent each scan-plane as a function of the slow scanning-mirror's position to form a "half-pipe". Curvature was achieved via rapid remote focussing synchronised with the scan pattern, as described in detail elsewhere[S2]. The degree of peak axial curvature was empirically adjusted between 0-150 µm between scans and planes to achieve best overall sampling of the entire brain.

Pre-processing and extraction of response amplitudes of 2-photon data. Recordings were linearly interpolated to 42 Hz and manually aligned between fish using a time-averaged brightness projection. Regions of interest (ROIs), corresponding to individual and/or small groups of neighbouring neuronal somata were defined automatically using custom Python scripts. In short, we used a "quality-index" (QI, described in detail elsewhere[S6]) to first identify individual pixels that exhibited reliable responses to repeated stimulation. For this,

we computed a pixel-wise QI-projection of the deinterleaved recording, sorting QI-pixels in descending order. The resulting curve was differentiated using *scipy.interpolate.splrep*. Pixel indices between inflections of the differential were projected back into space. Contours were identified using dilation (3,3)-erosion(2,2) and contour finding of Python-OpenCV. Individual contours were taken as ROIs, discarding any ROIs with a diameter > 15 µm. QI per ROI was then recalculated and used for further thresholding at QI>0.5. From here, fluorescence traces were extracted and z-normalized based on the 6 s at the beginning of recording prior to stimulus presentation. Overall, this strategy served to balance the need to combine multiple pixels into ROIs to boost their signal-to-noise, with a goal of keeping ROIs as small and localised as possible to approximately report the signals single, or from at most very small groups of somata that responded in a similar manner. This compromise was necessary to accommodate the large size of the scan pattern capturing the entire length of the brain while also maintaining a reasonable imaging rate. A stimulus time marker embedded in the recording data served to align the traces relative to the visual stimulus with a temporal precision of 1 ms.

Separation of On- and Off responses. Calcium traces were deconvolved using ARMA(1) (caiman.source_extraction.cnmf.deconvolution, Ref[S7]). Inferred discrete events were partitioned into events occurring during stimulus presentation and the complement.

Computing the brain's bulk spectral tuning functions. Inferred events were summed over respective stimulus time windows. Sums were averaged over all recorded traces. Contrast between On and Off portions of the response was calculated as their difference over their sum.

Natural Imaging Data Analysis. Hyperspectral data were obtained from Ref[S3] and element-wise multiplied with a deuterium light source derived correction curve (see online data). The data were restricted to the domain of 360-650 nm. Here, the long-wavelength end of the domain was decided based on the long-wavelength opsin absorption curve; the short-wavelength end was dictated by the sensitivity of the spectrometer. Spectra were scaled by standard deviation within a given scene. Traces were multiplied with the respective On- and Off-filters. The responses were summed within spectrum to produce a single number per point spectrum (or 800-long vector per scan). These vectors were standard-deviation-scaled within a scene. Spatial projections of filter responses were Gaussian-smoothed in space (σ=2px).

SUPPLEMENTAL REFERENCES

S1. Franke, K., Berens, P., Schubert, T., Bethge, M., Euler, T., and Baden, T. (2017). Inhibition decorrelates visual feature representations in the inner retina. Nature *542*, 439–444.

S2. Janiak, F.K., Bartel, P., Bale, M., T, Y., Komulainen, E.H., Zhou, M., Staras, K., Prieto-Godino, L.L., Euler, T., Maravall, M., *et al.* (2019). Divergent excitation two photon microscopy for 3D random access mesoscale imaging at single cell resolution. bioRxiv, 821405.

S3. Zimmermann, M.J.Y., Nevala, N.E., Yoshimatsu, T., Osorio, D., Nilsson, D.-E., Berens, P., and Baden, T. (2018). Zebrafish Differentially Process Color across Visual Space to Match Natural Scenes. Curr. Biol. *28*, 2018-2032.e5.

S4. Dunn, T.W., Mu, Y., Narayan, S., Randlett, O., Naumann, E.A., Yang, C.T., Schier, A.F., Freeman, J., Engert, F., and Ahrens, M.B. (2016). Brain-wide mapping of neural activity controlling zebrafish exploratory locomotion. Elife.

S5. Yoshimatsu, T., Bartel, P., Schröder, C., Janiak, F.K., St-Pierre, F., Berens, P., and Baden, T. (2020). Near-optimal rotation of colour space by zebrafish cones in vivo. bioRxiv.

S6. Baden, T., Berens, P., Franke, K., M, R.R., Bethge, M., and Euler, T. (2016). The functional diversity of retinal ganglion cells in the mouse.

S7. Giovannucci, A., Friedrich, J., Gunn, P., Kalfon, J., Brown, B.L., Koay, S.A., Taxidis, J., Najafi, F., Gauthier, J.L., Zhou, P., *et al.* (2019). CaImAn an open source tool for scalable calcium imaging data analysis. Elife.

Author contributions

P.B. and T.B. designed the study, with input from F.K.J. and D.O. P.B. performed 2-photon data collection, pre-processing, and analysis. P.B. built the light-stimulator with input from F.K.J., who also built the volumetric mesoscale two-photon system. P.B. performed natural imaging data analysis, with input from T.B. and D.O. T.B. wrote the manuscript with inputs from all authors.
